# A Six-Fold Symmetric Metamaterial Absorber

**DOI:** 10.3390/ma8041590

**Published:** 2015-04-03

**Authors:** Humberto Fernández Álvarez, María Elena de Cos Gómez, Fernando Las-Heras

**Affiliations:** Área de Teoría de la Señal y Comunicaciones, Dpt. Ingeniería Eléctrica, Universidad de Oviedo, Edificio Polivalente, Mod. 8, Gijón 33203 (Asturias), Spain; E-Mails: medecos@uniovi.es (M.E.C.G.); flasheras@uniovi.es (F.L.-H.)

**Keywords:** metamaterial absorber (MMA), double-negative media (DNM), negative refractive index (NIR), left handed media (LHM), polarization-insensitive, angular stability

## Abstract

A novel microwave metamaterial absorber design is introduced along with its manufacturing and characterization. Significant results considering both bandwidth and angular stability are achieved. Parametric analysis and simplified equivalent circuit are provided to give an insight on the key elements influencing the absorber performance. In addition, the constitutive parameters of the effective medium model are obtained and related to the absorber resonant behavior. Moreover, a new thinner and more flexible absorber version, preserving broad bandwidth and angular insensitive performance, is simulated, and an 8 × 8 unit-cells prototype is manufactured and measured for a limited angular margin in an anechoic chamber.

## 1. Introduction

Metamaterial absorbers MMAs are interesting structures focusing the attention of many researchers from a few years ago [1,2,3,4,5,6,7,8,9,10,11,12]. These structures have properties not found in nature and could be characterized as homogeneous media through their macroscopic parameters [1]. Tuning both magnetic permeability μ and electrical permittivity ε makes it possible to shift the resonance frequency and vary the MMA bandwidth.

There are lots of studies based on MMAs with different applications such as antennas RCS reduction [13], sub-diffracting imaging [14], invisibility cloaks [15], and so on. One of the most promising applications of these absorbers is the use of MMA as an electromagnetic sensor to detect different materials (explosive, drugs, *etc.*) owing to the change of the resonance frequency [16,17]. Moreover, the use of metamaterials in active tunable applications is spread in the recent years, above all at terahertz frequencies [18,19,20,21,22].

This paper aims to design a resonant MMA exhibiting near unity absorption along a wide bandwidth, under different incident angles and polarizations. However, it is rather difficult to obtain an absorber with polarization [23] and incidence angle insensitive performance [24,25] while preserving a broad bandwidth.

Most of the MMAs found in literature are based on unit-cells with a four-fold rotational symmetry [6,8,13,16,17,18,19,20,21,22,23,24,26,27,28]. In contrast, a novel unit-cell geometry with six-fold rotational symmetry is presented in this paper. Furthermore, the constitutive parameters of the resulting periodic structure are studied to improve its behavior as absorber, regarding bandwidth and angular stability. Once the design is fixed, it is adjusted to be manufactured in a more flexible and thinner dielectric substrate. Finally, it is measured and some conclusions are obtained.

## 2. Results and Discussion

### 2.1. Metamaterial Absorber Design and Characterization

The absorption of a structure can be calculated as A(ω) = 1 − R(ω) − T(ω), where A(ω), R(ω) and T(ω) are respectively the frequency dependent absorption, reflection and transmission. The reflection can be reduced by matching the structure impedance to the free space one (1).

(1) $$\text{Z(ω)}=\sqrt{\frac{\text{ε}\left(\text{ω}\right)}{\text{μ}\left(\text{ω}\right)}}=\text{120π}$$

**Figure 1:**
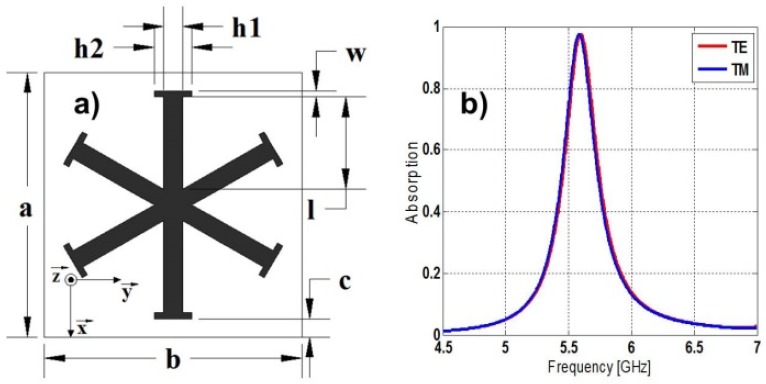
(**a**) Unit-cell geometry; (**b**) absorption for TE and TM polarization.

The proposed unit-cell consists of six-fold symmetry metallization geometry on its top side (Figure 1a), which makes the structure more stable in terms of incident field polarization [29] and a fully metallic back plate to minimize the transmission. Copper of 35 μm thickness is used for the metallic parts whereas FR4 dielectric with relative permittivity ε*_r_ =* 4.1, thickness *d* = 1 mm and loss tangent tanδ = 0.025 is fixed between both metallization layers. This dielectric is commonly used by most authors [3,4,5,6,7,8,9,10,13,30,31,32,33,34,35], which makes possible to show the advantages of the presented design through comparison.

The commercial software Ansoft HFSS is used to carry out the 3D electromagnetic simulations. The simulation setup is based on a unit-cell along with Floquet Ports and Master/Slave periodic boundary conditions (PBCs) applied to mimic an infinite structure. The TE and TM polarizations of the incident plane-wave are chosen in such a way that the electric field is oriented along y and x direction, respectively (see Figure 1).

The initial unit-cell dimensions proposed are as follows: a = 14.372 mm, b = 13.74 mm, l = 5 mm, h1 = 1 mm, h2 = 2 mm, c = 1 mm and w = 0.32 mm. Varying these parameters it is possible to shift the resonance frequency along a wide frequency band.

Absorption values of 97.62% at 5.599 GHz and 97.45% at 5.583 GHz are obtained in simulation under normal incidence, respectively for TE and TM polarized plane-waves. The bandwidth values at half maximum peak absorption (FWHM) are respectively 5.55% and 5.53% for each polarization (see Figure 1b). These results outperform most reported in the literature for MMAs based on a single expanded unit-cell (see Section 2.4).

**Figure 2:**
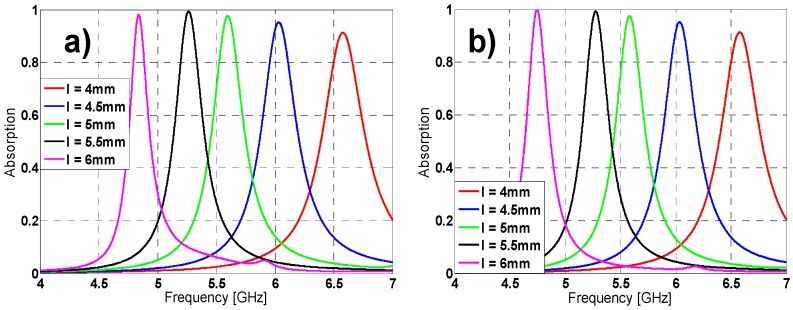
Results in simulation for different lengths of *l* for both (**a**) TE (**b**) TM.

**Figure 3:**
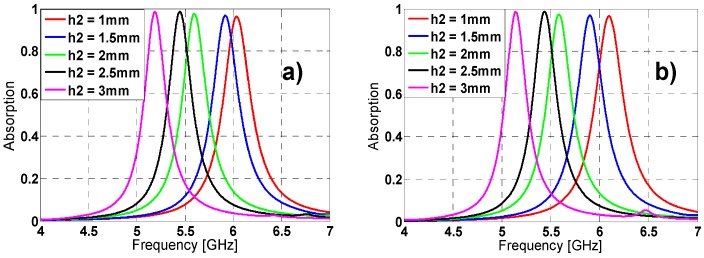
Absorption results in simulation for different lengths of h2 for both (**a**) TE (**b**) TM.

**Figure 4:**
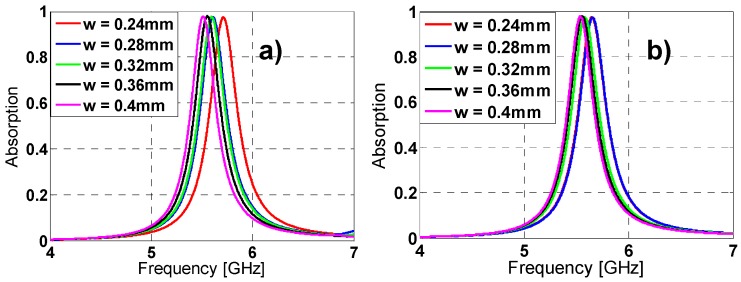
Absorption results in simulation for different lengths of *w* for both (**a**) TE (**b**) TM.

The tuning of some unit-cell parameters makes possible to vary the resonance frequency, absorption peak and bandwidth (see Figure 2, Figure 3 and Figure 4). From Figure 2, by increasing “l” length the resonance frequency (f_r_) and the bandwidth decrease, while the absorption is improved. The same behavior is observed in Figure 3 and Figure 4 for variations in h2 length and w width. This performance makes sense considering that this kind of materials wavelength is roughly proportional to their “unwound” copper length L (λ_0_ ~ 2L) [26]. It is noticed that combining the “l”, “h2” and “w” parameters it is possible to achieve either a fine or a rough adjustment in the resonance frequency value.

### 2.2. Equivalent Circuit Model

**Figure 5:**
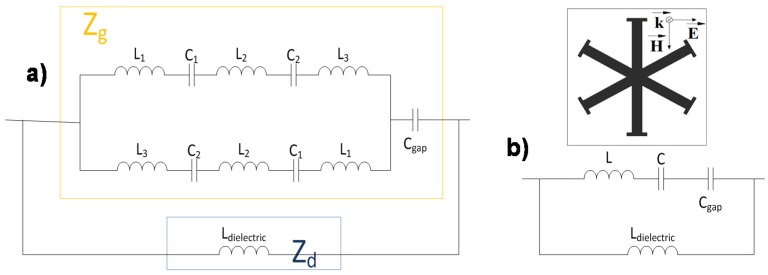
TE polarization: (**a**) Equivalent circuit; (**b**) Simplified circuit.

To better understand the resonance behavior of the MMA structure an equivalent circuit model is proposed under plane-wave incidence. The impedance seen by an obliquely incident plane wave can be described, provided that the unit-cells under study are electrically small in terms of periodicity, as a parallel connection of the effective grid impedance Z_g_(ω) concerning the metal-dielectric periodic pattern and the input impedance of the grounded dielectric substrate Z_d_(ω), both of them frequency dependent [36]. Following this idea, equivalent circuit models for both TE and TM polarization are devised. The equivalent inductances and capacitances on the grid impedance vary depending on the field polarization angle ϕ [37]. As an example for ϕ = 0° in the TE case (see Figure 5) to obtain the grid impedance there are a series of inductances and capacitances on the upper part of the structure (L_1_, C_1_, L_2_, C_2_ and L_3_) which are owing to the tips of the branches and the gap between each branch, respectively and on the lower part, there are the same series of the inductances and capacitances but with the opposite distribution owing to the variation of the field regarding the symmetries. The other key element involved on the grid impedance is the capacitance (C_gap_) between adjacent unit-cells. The input impedance of the grounded dielectric substrate Z_d_(ω) can be modeled as an inductance (L_dielectric_) which is the one corresponding to the dielectric backed by a metallic plate.

**Figure 6:**
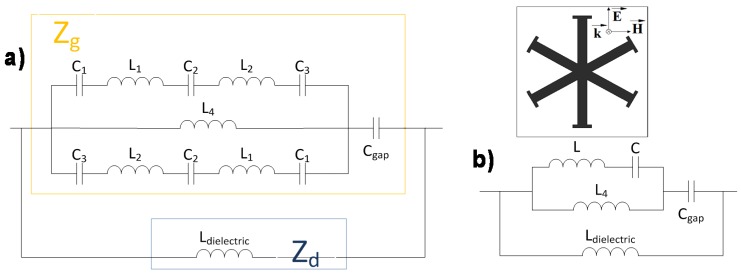
TM polarization: (**a**) Equivalent circuit; (**b**) Simplified circuit.

For the TM polarization (see Figure 6), there is a distribution of capacitances and inductances disposed in series (C_1_, L_1_, C_2_, L_2_ and C_3_) that are the result of the gap between branches and the tips of the branches, similar to the TE case but in this one, the orientation of the field is different and for that reason these fields “see” a different distribution of capacitances and inductances. Under normal incidence (θ = 0°) the Z_d_(ω) impedance for TM polarization is identical to the one defined for TE polarization so that it can also be modeled by an inductance (L_dielectric_). However, under oblique incidence Z_d_(ω) is additionally affected by the incidence angle [37].

It is important to point out that these equivalent circuits are only a simple approximation aiming to explain the resonant behavior and for better understanding the influence of the unit-cells geometry elements on the angular stability. The dielectric losses are considered negligible in this sense.

By applying basic circuits theory one can arrive to the simplified circuits shown on Figure 5b and Figure 6b. From the results obtained in these aforementioned simplified circuits, the structure can be characterized as a parallel LC resonator, considering that the metal-backed dielectric inductance and the capacitance among unit-cells are the main parameters in the equivalent circuit. This circuit has a quality factor $\left(Q\text{​}=\sqrt{C/L}\right)$ which is inversely proportional to the bandwidth $\left(BW\text{​}=f_{r}/Q\right)$. Therefore, it is concluded that the higher the inductance, the broader the bandwidth obtained.

### 2.3. Angular Stability

**Figure 7:**
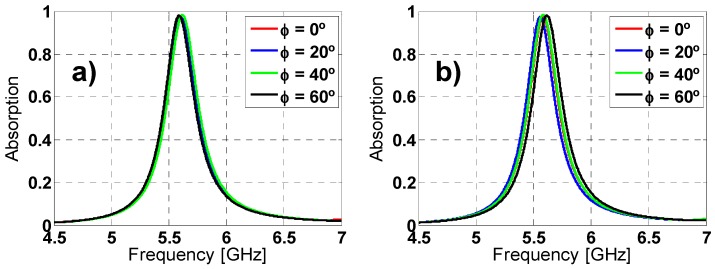
Absorption simulation results for different polarization angles (ϕ) of the incident field for both (**a**) TE and (**b**) TM polarizations.

**Figure 8:**
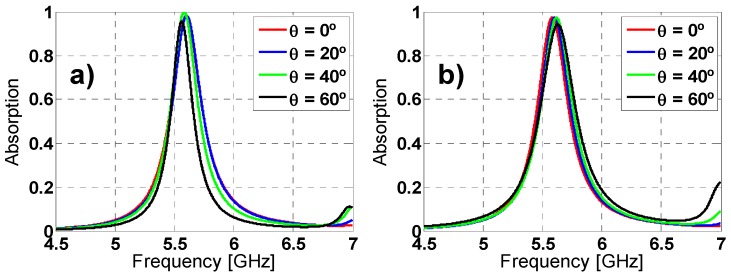
Absorption simulation results under different incident angles (θ) for both (**a**) TE and (**b**) TM polarizations.

Other important characterization is the analysis of the angular stability for different polarizations and incidence angles. For that purpose, on one hand the polarization angle of the incident field (ϕ) has been varied from 0° to 60° (due to its symmetry, since the remainder angles (60°–360°) can be extrapolated) in steps of 20° for both TE and TM polarized incident plane-wave under normal incidence (θ = 0°). On the other hand, the incidence angle (θ) has been also varied from 0° to 60° for both TE and TM polarizations; the results are depicted in Figure 7 and Figure 8, respectively. For ϕ variations the resonance frequency almost remains unaltered for TE polarization. However, for TM polarization the resonance frequency undergoes a small shift. The resonance frequency, the bandwidth and the maximum absorption peak are negligible affected by the θ variations. These stable results are owing to the symmetry and the equivalent inductance of the unit cell [27,37].

### 2.4. Comparison with the State of the Art

The aim of this subsection is to compare the prototype proposed with the current state of the art. To make a fairly comparison it is necessary that all metamaterials have the same dielectric (FR4). This comparison is shown in the Table 1. It is clear that the overall characteristics of this prototype outperform the ones encountered in the literature.

**Table 1:** Metamaterial absorber (MMA) comparison.

Prototype	Resonant Frequency [GHz]	Thickness * [mm]	Electrical Thickness	FWHM (simulation) [%]	Polarization Insensitive	Angle of incidence insensitive
Paper	5.599	1.07	λ_fr_/50	5.55	Yes	Yes
[3]	11.65	0.737	λ_fr_/34.94	4	No	Till 16°
[13]	5.57	0.57	λ_fr_/94.49	3.9	Yes	Yes but worse than in this paper
[30]	9.5	1.034	λ_fr_/30.54	3.79	Yes	Yes but worse than in this paper
[31]	10.05	0.76	λ_fr_/39	4.8	Yes	Yes but worse than in this paper
[32]	10.14	1	λ_fr_/29.586	4.7	Yes	Yes
[33]	11.3	0.418	λ_fr_/63	4.2	Yes	Till 25°
[34]	8.10	1.07	λ_fr_/34.61	4.68	No	No
[35]	10	0.87	λ_fr_/34.48	4.9	Yes	Yes

* The thickness takes into account the thicknesses of both the dielectric and the copper metallization layers.

There are other works based on six-fold unit cells [33,34,35,36,37,38]. The unit cell proposed in [38] is based in an interdigitalized unit-cell. The authors claimed that the prototype is polarization insensitive and the results from 0° to 90° rotational angles are shown. However, the unit-cell does not have six-fold symmetry and the insensitivity for other polarization angles greater than 90° will be not achieved. That means that the polarization at 20° and the one at 340°, for example, will not coincide and it could be concluded that it is not polarization insensitive for the whole range of angles (from 0° to 360°). Moreover, it is said that the FWHM is 11%, nevertheless there are two clear peaks at 9.8 and 10.3 GHz with a valley between them with an absorption of 55% at 10 GHz. Therefore, it is not true that the absorber can work properly in the complete frequency range. In addition, there is no analysis of the behavior of the prototype for different incident angles. Finally, the electrical thickness of this unit cell is the same as the proposed in this paper.

The unit cell proposed in [33] suffers from the same problem in the polarization than the previous paper. This cell is electrically thicker than the cell proposed in this paper and the bandwidth is narrower. Moreover, there is no analysis of the prototype behavior under different incidence angles. 

### 2.5. Flexible Metamaterial

This work also aims to achieve a more flexible MMA, adaptable to different rough surfaces. For that purpose, the MMA dielectric was replaced by a thinner and more conformable one. Table 2 shows the considered dielectrics and their properties which foresee the changes in the resonance frequency, bandwidth and absorption peak owing to the variations experimented by relative permittivity, loss tangent and thickness compared to FR4 dielectric.

**Table 2:** Flexible dielectrics tested.

Dielectric	ε_r_	tanδ	Thickness (mm)
Arlon 25n	3.28	0.0025	0.457
RO3003	3	0.0013	0.800
RO4003C	3.38	0.0027	0.203
RO4003C	3.38	0.0027	0.406

**Figure 9:**
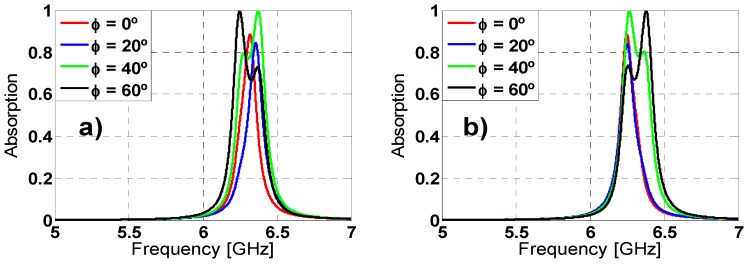
Absorption simulation results for different polarization angles (ϕ) of the incident field for both (**a**) TE and (**b**) TM polarizations.

**Figure 10:**
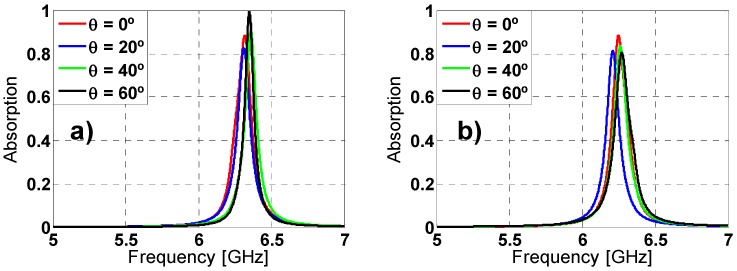
Absorption simulation results under different incident angles (θ) for both (**a**) TE and (**b**) TM polarizations.

Once the MMA was simulated with all of the proposed dielectrics and several thicknesses, the best absorption results were obtained for Arlon 25n dielectric with only one 0.457 mm thickness layer and 18 μm thickness copper sheets. The results obtained under normal incidence have maximum absorption peak of 88.35% at 6.319 GHz and 88.25% at 6.249 GHz for TE and TM polarization, respectively. The bandwidth is 2% at FWHM for both TE and TM polarizations. These results attract attention considering that the maximum absorption peak and bandwidth are smaller than the obtained with FR4 but it is necessary to pay attention to Table 3 which shows that the electric thickness of the MMA with Arlon 25n reduces the one of FR4 dielectric by a half. Moreover, the thickness presented here are thinner than the one presented in other documents [10]. The behavior under different polarizations at normal incidence has been analyzed for the new dielectric and the results are depicted in Figure 9. The incidence angle (θ) has been also varied from 0° to 60° for both TE and TM polarizations (see Figure 10). The angular stability for TM polarization is slightly reduced compared to FR4 prototype (due to the reduction of ε_r_ [37]) but in any case it can be considered stable.

**Table 3:** MMA thickness.

Dielectric	Polarization (f_r_ [GHz])	Thickness (mm)	Electric thickness
FR4	TE (5.599)	1.07	~λ_r_/50
FR4	TM (5.583)	1.07	~λ_r_/50
Arlon 25n	TE(6.319)	0.493	~λ_r_/96.26
Arlon 25n	TM(6.249)	0.493	~λ_r_/97.33

### 2.6. Constitutive Parameters of Metamaterial Absorber

**Figure 11:**
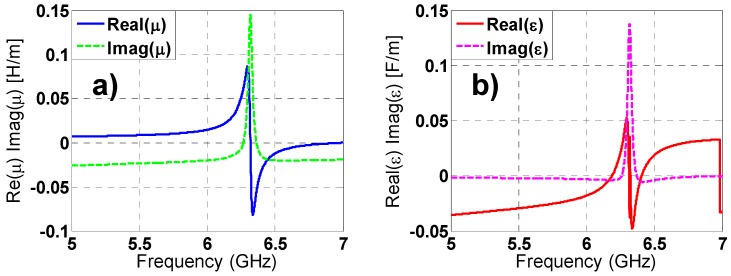
TE polarization: (**a**) real and imaginary parts of magnetic permeability; (**b**) real and imaginary parts of electric permittivity.

**Figure 12:**
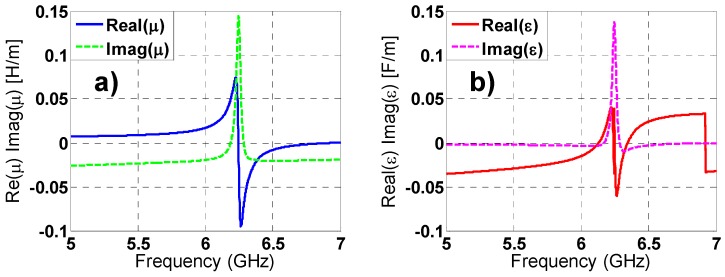
TM polarization: (**a**) real and imaginary parts of magnetic permeability; (**b**) real and imaginary parts of electric permittivity.

As regards classical crystals states a medium can be considered homogenous if the following conditions are satisfied: The dimensions of the elementary cell must be much smaller than the incident wavelength and the sample must contain a large number of elementary cells. Satisfying the previous considerations, a MMA can be characterized as an effective medium through its constitutive parameters: Complex electric permittivity ε(w) = ε_1_(w) + jε_2_(w) and a complex magnetic permeability μ(w) = μ_1_(w) + jμ_2_(w) that gives an idea of the structure performance. There is a distinct correspondence between constitutive parameters and S-parameters following the Ziolkowski’s work [28]. The results obtained applying this theory (see Figure 11 and Figure 12) for both TE and TM polarizations, are in good agreement with the frequency-domain absorption simulation results, considering that at the resonance frequency both ε(w) and μ(w) have a resonant behavior and the imaginary parts, which are directly connected with the losses in the MMA, achieve a maximum value.

## 3. Experimental Section

**Figure 13:**
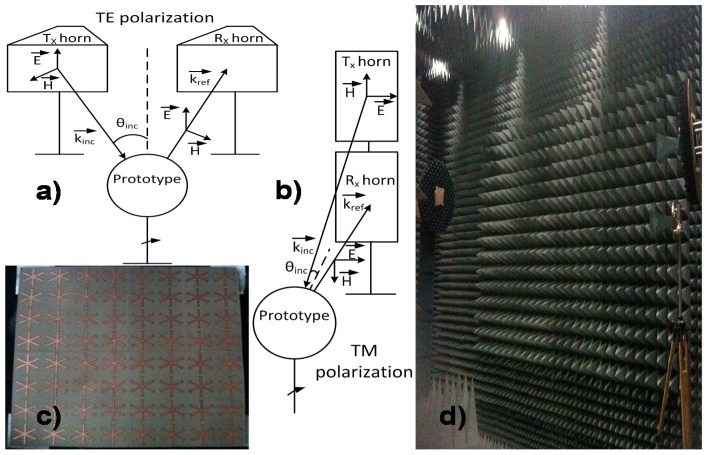
Measurement setup configurations: (**a**) TE; (**b**) TM; (**c**) manufactured prototype; and (**d**) anechoic chamber set up.

There are several points to take into consideration before choosing the prototype dimensions. It is obvious that the bigger the prototype, the fewer differences with the simulated infinite one exist, but the narrower the main lobe in the scattered field pattern and fewer angles of incidence can be measured. Another important consideration is the limitations of the manufactured machine regarding the maximum prototype size that the machine allows fabricating. Hence, getting into account the previous considerations a trade-off solution has been adopted: An 8 × 8 unit-cells (Figure 13c) prototype is manufactured using laser micromachining (LPKF’s ProtoLaserS). The measurements for different polarization and incidence angles of the MMA’s reflection coefficient have been carried out in an anechoic chamber (Figure 13d). Figure 13a,d show the arrangement of the horns antennas regarding the prototype for TE and TM measurements, respectively.

To obtain the absorption values it is necessary to measure, as reference, a metallic plate with the same MMA prototype dimensions. The measurements are taken in the far-field region to ensure that a plane-wave impinges in the MMA [39] and to avoid near-field distortion.

**Figure 14:**
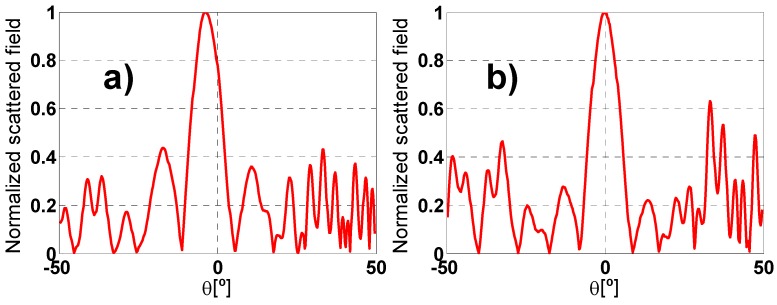
Scattered field pattern in anechoic chamber at 6.3 GHz: (**a**) TE MMA and (**b**) TM metallic sheet.

The scattered field pattern (see Figure 14) is calculated and the limitations, owing to the fact that the manufactured prototype is finite, are taken into account to analyze the results. Since the scattered field from the prototype and the metallic plate for the same polarization and arrangement of the set-up has the same shape in the two cases, but only the levels are different, no matter which scattered field is retrieved to get an idea of where the nulls, the main lobe and the secondary lobes are.

According to Figure 14a an angular margin of θ = ±8° can be measured for TE polarization and ϕ = 0°. Moreover, at θ = ±14° there is a secondary lobe in the scattered field pattern with enough dynamic range to obtain a clear absorption peak. Nonetheless, the scattered field from θ = +8° to θ = +14° and from θ = −8° to θ = −14° is very low, resulting in intelligible absorption peaks. For TM configuration an angular margin of θ = ±6° and a couple of values at θ = ±14° can be measured. The fact that the measure for θ = 14 is “better” than the one obtain for θ = 6 in TM polarization is mainly due to the more significant value in the scattering pattern for θ = 14°. The smaller angular margin in TM configuration is owing to TE and TM polarizations having different scattered field pattern as it can be seen in the Figure 14.

**Figure 15:**
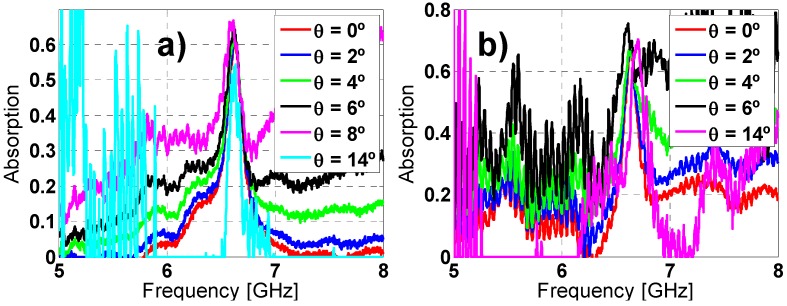
Absorption measurement results for different incident angles (θ) for both (**a**) TE; and (**b**) TM polarizations.

The measurement results (Figure 15) show a resonance frequency shift and a lower absorbance peak level compared with the simulated ones. This is due to the finite prototype dimensions, the manufacturing tolerances, the diffraction on edges and other scatterings in the anechoic chamber. However, these results are in accordance with the ones presented in literature.

Throughout the literature many works can be found conducting electromagnetic simulations of MMAs in the Microwaves frequency range in similar conditions as those presented in this paper. This means, under plane-wave incidence and so considering far-field conditions. However, when analyzing in detail the sections devoted to measurements in anechoic chamber, one can realize that far-field condition are not met considering the size of the prototypes and the distance at which the measurements are performed. Therefore, the measurements are not valid to compare with simulations and, moreover, it is not justified theoretically nowadays that transformation of such near-field measurements to far-field can be realized in such conditions. The scattered field pattern *versus* the incidence angle (θ) obtained in anechoic chamber for a metallic plate with the same dimensions of the MMA to be characterized, shows a main lobe and several secondary lobes with nulls (*i.e.*, very small field amplitude) between them. The MMA exhibits lower field levels on the pattern lobes and nulls in the same positions as the metallic plate, so that for many incident angles it is not possible to perform a measurement.

## 4. Conclusions

A novel six-fold symmetric MMA has been designed. A parametric characterization revealing its flexible performance in the band from 4 to 7 GHz has been conducted. The equivalent circuit model for TE and TM polarization has been obtained to give an insight on how to improve the bandwidth. The satisfactory results in both angular stability and bandwidth have been shown for both TE and TM polarization. In that point it is necessary to realize the difficulties to get at the same time good angular stability and wider bandwidth (above all at the frequencies addressed in this paper since the lower frequency, the more complicated is to get a wider bandwidth owing to the LC behavior of these structures discussed before).

Furthermore, the resonance phenomenon has been explained, obtaining the constitutive parameters according to the Ziolkowski’s work for effective medium model. Besides, a thinner and more flexible MMA has been designed keeping excellent characteristics for both angular stability and bandwidth. The measurements results are in reasonable accordance with the simulations ones bearing in mind the shortcomings restraining the measurement process. 

Possible future applications on the actively tuneable behavior of this MMA adding some kind of resistances (potentiometers, NTC or PTC resistances and so on) are left to future developments.
